# Development and validation of a clinical rule for the diagnosis of chikungunya fever in a dengue-endemic area

**DOI:** 10.1371/journal.pone.0279970

**Published:** 2023-01-06

**Authors:** Raquel Pereira Batista, Yara Hahr Marques Hökerberg, Raquel de Vasconcellos Carvalhaes de Oliveira, Sonia Regina Lambert Passos

**Affiliations:** 1 Escola Nacional de Saúde Pública Sergio Arouca, Fundação Oswaldo Cruz, Rio de Janeiro, Rio de Janeiro, Brazil; 2 Hospital Universitário Clementino Fraga Filho, Universidade Federal do Rio de Janeiro, Rio de Janeiro, Rio de Janeiro, Brazil; 3 Laboratório de Epidemiologia Clínica, Instituto Nacional de Infectologia Evandro Chagas, Fundação Oswaldo Cruz, Rio de Janeiro, Rio de Janeiro, Brazil; 4 Faculdade de Medicina, Universidade Estácio de Sá (UNESA), Rio de Janeiro, Brazil; Federal University of São João del-Rei: Universidade Federal de Sao Joao del-Rei, BRAZIL

## Abstract

Rio de Janeiro is a dengue-endemic city that experienced Zika and chikungunya epidemics between 2015 and 2019. Differential diagnosis is crucial for indicating adequate treatment and assessing prognosis and risk of death. This study aims to derive and validate a clinical rule for diagnosing chikungunya based on 3,214 suspected cases consecutively treated at primary and secondary health units of the sentinel surveillance system (up to 7 days from onset of symptoms) in Rio de Janeiro, Brazil. Of the total sample, 624 were chikungunya, 88 Zika, 51 dengue, and 2,451 were negative for all these arboviruses according to real-time polymerase chain reaction (RT-qPCR). The derived rule included fever (1 point), exanthema (1 point), myalgia (2 points), arthralgia or arthritis (2 points), and joint edema (2 points), providing an AUC (area under the receiver operator curve) = 0.695 (95% CI: 0.662–0.725). Scores of 4 points or more (validation sample) showed 74.3% sensitivity (69.0% - 79.2%) and 51.5% specificity (48.8% - 54.3%). Adding more symptoms improved the specificity at the expense of a lower sensitivity compared to definitions proposed by government agencies based on fever alone (European Center for Disease Control) or in combination with arthralgia (World Health Organization) or arthritis (Pan American Health Organization, Brazilian Ministry of Health). The proposed clinical rule offers a rapid, low-cost, easy-to-apply strategy to differentiate chikungunya fever from other arbovirus infections during epidemics.

## Introduction

Chikungunya fever is a neglected arbovirus infection that continues to spread throughout the world, affecting up to 1 billion people [1]. The clinical presentation of chikungunya is similar to that of other arbovirus infections such as dengue or Zika [2, 3]. Symptomatic chikungunya (CHIK) infections present mostly with high fever, headache, exanthema, myalgia, and severe joint pain [4–6]. The disease can evolve in three phases, namely acute, subacute, and chronic [7], the latter accounting for 59% of cases [8]. The high burden of chikungunya fever varies from 427 to 1,407 years with disability, and 385,835 to 429,058 individuals can develop chronic inflammatory rheumatism after CHIK infection in endemic areas of Latin American countries [7, 9].

Chikungunya virus was detected in 2013 in Latin America [10, 11] and has predominated mainly in urban areas of dengue-endemic countries such as Rio de Janeiro, the second largest Brazilian city [12–15]. Brazil reported more than 1.3 million probable cases from 2016 to 2019, most of which in the Southeast, with an incidence of 511.5 and 104.6 per 100 thousand inhabitants, respectively, in 2016 and 2019 [16, 17].

The reported cumulative annual incidence rates for chikungunya in Rio de Janeiro state, in Southeast Brazil, were 105.1/100,000 in 2016 and 492.8 in 2019 [15], mainly in the state capital [12]. Spatial overlap between dengue, Zika, and chikungunya [12], detected in the city of Rio de Janeiro between 2015 and 2019, poses a challenge for differential diagnosis, especially during outbreaks. Such differential diagnosis is crucial for promptly determining adequate clinical management and prognosis as well as for monitoring the effectiveness of potential preventive and therapeutic interventions [18]. Clinical prediction rules based on two or more clinical or unspecific laboratory predictors are useful for guiding daily decisions by health professionals [19, 20], thereby improving prognosis. The rules can also be used as a diagnostic tool to detect cases promptly for surveillance purposes.

The Brazilian Health Surveillance Guidelines of 2017 [21] proposed a differential clinical diagnosis between chikungunya, dengue, and Zika to orient health professionals. However, although many studies proposed clinical rules for diagnosing dengue [22–27] and Zika [23, 28], almost none investigated chikungunya fever [29]. The current study thus aimed to derive and validate a clinical rule for chikungunya diagnosis based on a large sample of outpatients seen in the public healthcare system in the city of Rio de Janeiro, where dengue and Zika are endemic.

## Materials and methods

This was a cross-sectional diagnostic study of all adult patients consecutively seen for arbovirus infections in healthcare units of the RT-qPCR sentinel surveillance system in the city of Rio de Janeiro. The study followed the Transparent Reporting of a multivariable prediction model for Individual Prognosis or Diagnosis (TRIPOD) Statement for clinical prediction models [19], complemented by the Standards for Reporting of Diagnostic Accuracy Studies (STARD) [30]. These guidelines propose a checklist to improve the transparency of reports on prediction and accuracy studies, allowing an appraisal of risks of bias and applicability of study results [19, 30].

From January 2016 to September 2019, trained nurses recruited patients with clinical suspicion of arbovirus infections consecutively seen at 23 public healthcare units (primary and urgent/emergency care) in the sentinel surveillance system. Cases were eligible if they reported or presented fever (axillary temperature > 38ºC) or exanthema up to 7 days with at least two of the following symptoms: headache, retro-orbital pain, myalgia, arthralgia, prostration, conjunctivitis, nausea, vomiting, and limb edema, after ruling out bacterial infections such as tonsillitis, sinusitis, or pneumonia. After evaluation by a physician, patients provided urine and blood samples, the latter centrifuged and stored at 2°C to 8°C at the local level. Biological samples were referred within 24 hours to reference laboratories of two institutions, the Evandro Chagas National Institute of Infectious Diseases of the Oswaldo Cruz Foundation (2016–2018) and the Noel Nutels Central Laboratory of Rio de Janeiro State (2018–2019). One-step real-time polymerase chain reaction (RT-PCR) was the gold standard for defining chikungunya, dengue (serotypes DENV-1 to DENV-4), Zika (in serum and urine), or negative status, following the manufacturer’s instructions (ZDC Molecular Kit, BioManguinhos, Fiocruz) [31].

Individual data were reported to the Rio de Janeiro Information System on Diseases of Notification (SINAN-Rio), a database available upon formal authorization and ethical approval. Clinical and sociodemographic data were collected. New variables were generated based on the case definitions by the following agencies: a) World Health Organization WHO 2015 [32]: fever and arthralgia; b) Pan American Health Organization or Centers for Disease Control PAHO/CDC 2011 [7]: fever and severe arthralgia or arthritis; c) Brazilian Ministry of Health, 2017 [21]: fever and arthralgia or arthritis; and the European Centre for Disease Control ECDC 2018 [33]: fever in persons living in or traveling to endemic regions.

A complete case data analysis was performed in R software, version 3.6.1 [34]. Descriptive statistics were absolute and relative frequencies (with the respective 95% confidence intervals for proportions) of categorical variables according to chikungunya status. The study used random split-half samples. In the first random half (sample 1), single covariate and multiple binary logistic regression models were performed to derive a clinical rule for diagnosing chikungunya. The first multiple regression model included all clinical predictors with p < 0.2 in the simple regressions. The final multiple regression model included variables with p < 0.05 in Wald test statistic, adjusted by days since onset of symptoms (≤ 3 and 3–7 days). Crude and adjusted odds ratios (OR) with 95% confidence intervals (95% CI) were reported. Goodness-of-fit of the final logistic regression models was compared with the Hosmer and Lemeshow test, the regression influence plot of studentized residuals, and hat values (Cook´s distance) [35, 36], besides the log-likelihood ratios between the full and null models. The Hosmer and Lemeshow test did not show lack of fit of the final multiple model and Cook´s distance did not show influence from observations. The score of the derived rule (“Rio rule”) was the weighted sum based on the beta coefficients (β) of the predictors included in the final model, rounded to the nearest upper integer value. We calculated the area under the receiver operating characteristic (AUC) curve (95% CI), and the optimal cut-off point was defined by the Youden index.

In the second random split-half sample (sample 2), we validated the derived clinical rule using the following accuracy parameters (95% CI): sensitivity, specificity, positive and negative predictive values, positive and negative likelihood ratios, and diagnostic odds ratios. Additionally, we compared the accuracy parameters of the validated clinical rule (Rio rule) with the parameters of the four case definitions mentioned previously (WHO 2015 [32], PAHO/CDC 2011 [7], ECDC 2018 [33] and Brazil 2017 [21]).

### Ethics statement

The study was approved by the Institutional Review Board (IRB) of the Brazilian National School of Public Health, Oswaldo Cruz Foundation, and authorized by the Rio de Janeiro Municipal Health Department (CAAE nº 16646719.6.3001.5279). This observational retrospective study used routinely collected surveillance data, fully anonymized before analysis. The IRB waived the need for informed consent.

## Results

**Fig 1:**
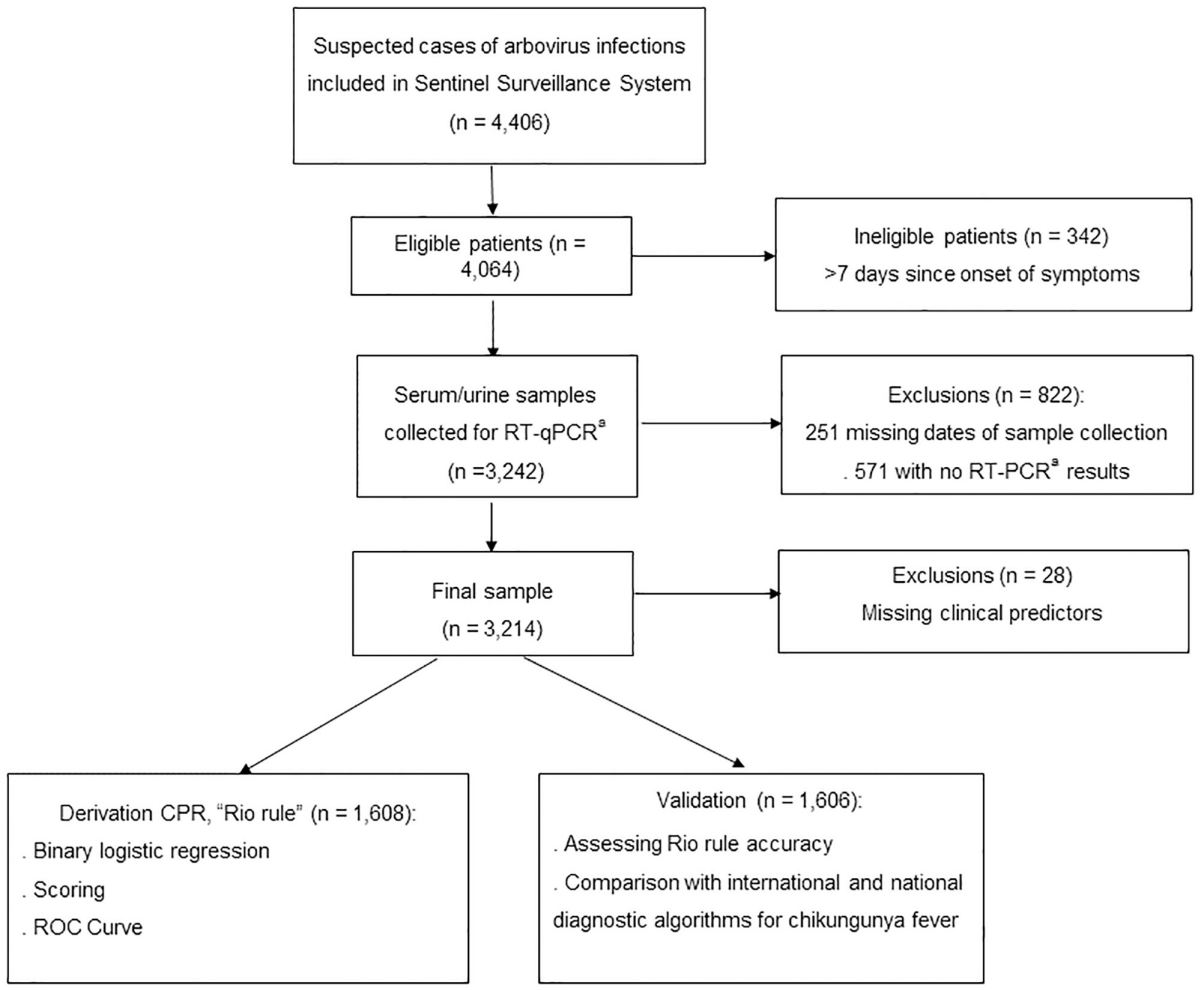
Flowchart of sample selection for derivation and validation of a clinical rule for chikungunya diagnosis. ^a^RT-PCR: real-time polymerase chain reaction (ZDC Molecular Kit, Fiocruz).

Of 4,406 patients with suspected arbovirus infections seen at the healthcare units, 3,242 (73.6%) met the eligibility criteria and provided serum or urine samples. This sample had similar age and gender distribution but a higher percentage of confirmed chikungunya cases (19.2% versus 14.2%) compared to the initial patient population. After excluding 28 (0.9%) patients with missing data for clinical predictors, the final study sample included 3,214 patients (Fig 1), most of whom living in the city of Rio de Janeiro, with black or brown race/skin color, low schooling, and up to 3 days since onset of symptoms (Table 1).

**Table 1:** Sociodemographic characteristics of suspected arbovirus cases, Rio de Janeiro, Brazil 2016–2019 (n = 3,214).

Variables		Chikungunya (N = 624)		Zika (N = 88)		Dengue (N = 51)		OFI ^a^ (N = 2,451)	
		N	% (95% CI)	n	% (95% CI)	n	% (95% CI)	n	% (95% CI)
Age (median)	(IQR) ^b^		38.0 (25.0 − 53.0)		28.0 (19.0–39.0)		30.0 (21.5–39.0)		30.0 (20.0–44.0)
Gender	Female	363	58.4 (54.4–62.3)	66	75.0 (64.6 − 83.6)	27	52.9 (38.5–67.1)	1382	57.0 (54.8 − 58.7)
	Male	259	41.6 (37.7–45.6)	22	25.0 (16.4–35.4)	24	47.1 (32.9 − 61.5)	1052	43.0 (41.2–45.2)
Schooling	Primary	285	61.9 (57.3–66.4)	30	57.7 (43.2 − 71.3)	11	45.8 (25.5–67.2)	944	60.1 (57.6 − 62.6)
	Secondary	142	30.9 (26.7–35.3)	16	30.7 (18.7–45.1)	9	37.6 (18.8 − 59.4)	468	29.8 (27.5–32.1)
	University	26	5.7 (3.7–8.2)	3	5.8 (1.2–15.9)	2	8.3 (1.0 − 26.9)	86	5.5 (4.4–6.7)
	Not applicable ^c^	7	1.5 (0.6–3.1)	3	5.8 (1.2–15.9)	2	8.3 (1.0 − 26.9)	72	4.6 (3.6–5.7)
Race/ ethnicity	Black/Brown	360	66.2 (62.0 − 70.1)	39	67.2 (53.6 − 78.9)	15	51.7 (32.5 − 70.5)	1069	60.0 (57.8 − 62.4)
	White	179	33.9 (29.0–37.0)	17	29.3 (18.1 − 42.7)	14	48.3 (29.4–67.5)	691	38.8 (36.6–41.2)
	Indigenous	4	0.7 (0.2 − 1.9)	─	─	─	─	4	0.2 (0.1–0.6)
	Asian descendant	1	0.2 (0.0 − 1.0)	2	3.4 (0.4 − 11.9)	─	─	15	0.8 (0.5 − 1.4)

95% CI: 95% confidence Interval;

^a^ OFI: other febrile illnesses;

^b^IQR: interquartile range;

^c^ children

**Fig 2:**
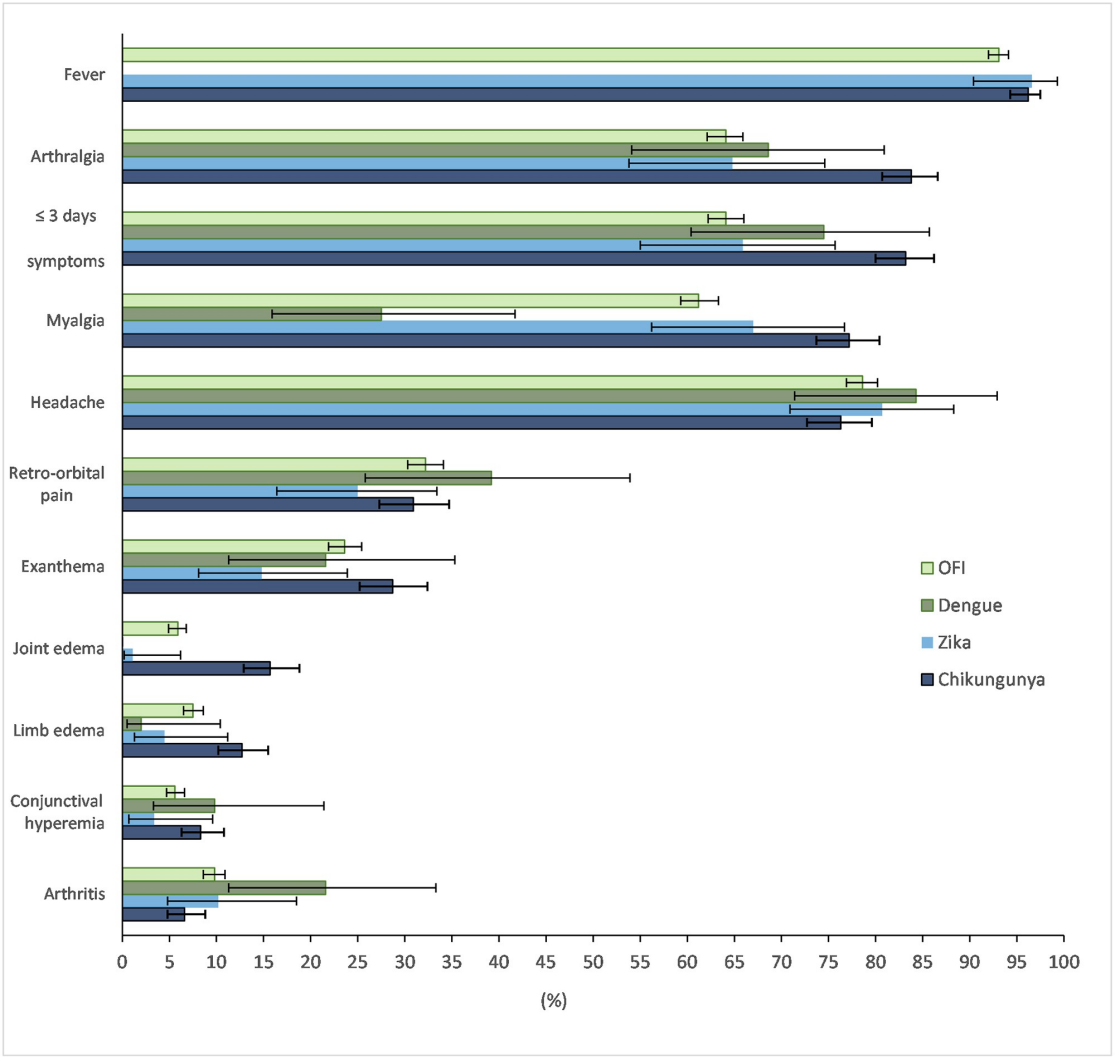
Presence of clinical signs and symptoms according to diagnosis, Rio de Janeiro, Brazil, 2016–2019 (n = 3,214). OFI: Other febrile illness.

The main clinical symptoms were fever, headache, arthralgia, and myalgia. Confirmed chikungunya cases were older on average than patients with the other arbovirus infections and other febrile illnesses (OFI) (Table 1). Chikungunya was the most frequent arbovirus infection, but most patients had other febrile illnesses. More than three-fourths of the chikungunya patients arrived at sentinel health units within three days of the onset of the disease with myalgia and arthralgia, while two-thirds of Zika cases arrived in the same time frame. Exanthema, myalgia, arthralgia, joint edema, and limb edema were more frequent in chikungunya cases than in other arbovirus infections or other febrile illnesses. Although common in chikungunya, the frequency of exanthema differed by less than 10% compared to dengue and other febrile illnesses (Fig 2 and S1 Table). Aphtha, lymphadenopathy, and neurological manifestations were rare in our sample (≤ 2%) (S1 Table).

**Fig 3:**
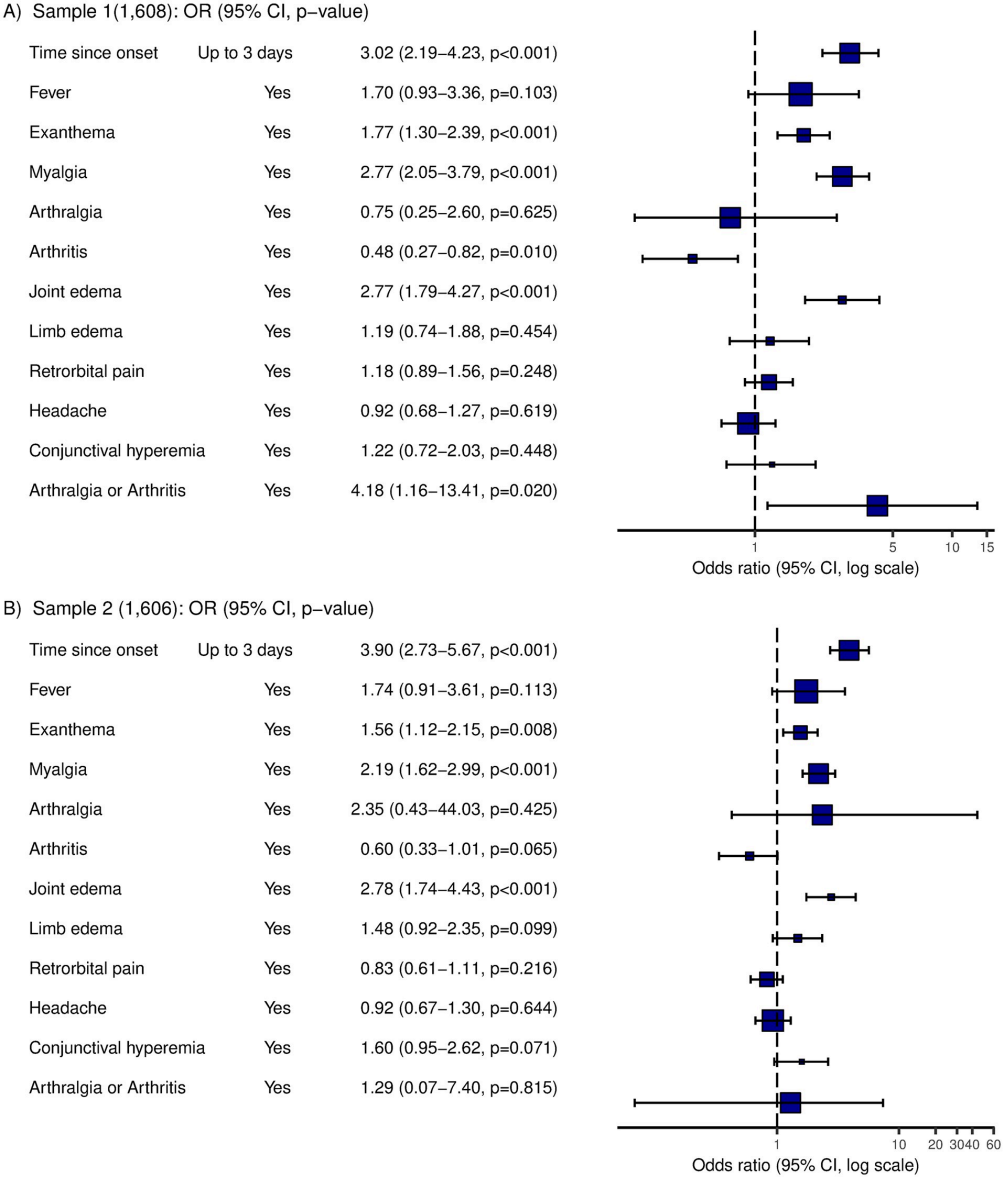
Odds ratio (OR) of clinical predictors according to chikungunya diagnosis (CHIK) in derivation (sample 1) and validation samples (sample 2). A. Sample 1 (1,608): OR (95% CI, p−value). B. Sample 2 (1,606): OR (95% CI, p−value).

The random split-half samples showed similar distribution of clinical predictors (Fig 3 and S2 Table). In the first random split-half sample (n = 1,608), the first multiple regression model included seven clinical predictors (p < 0.20 in simple regression models): fever, conjunctival hyperemia, exanthema, myalgia, arthralgia or arthritis, joint edema, and limb edema.

The final multiple model included five predictors: fever, exanthema, myalgia, joint edema, and arthralgia or arthritis, adjusted by days since onset of symptoms. Item weights varied from 1 to 2, and the score was obtained by weighted sum (Table 2). The Hosmer and Lemeshow test did not show lack of fit for the final multiple model, and Cook´s distance did not show influence from observations.

**Table 2:** Odds ratio (OR), β coefficients, and scores of clinical predictors for chikungunya diagnosis in the final multiple binary regression model*, sample 1 (n = 1,608).

Clinical predictors	Crude OR	(95% CI)	Adjusted OR	(95% CI)	β	Score
Fever	1.40	(0.77 − 2.72)	1.60	(0.87 − 3.14)	0.47	+1
Exanthema	1.47	(1.10 − 1.95)	1.80	(1.33 − 2.43)	0.59	+1
Myalgia	2.78	(2.08 − 3.77)	2.88	(2.14 − 3.93)	1.06	+2
Joint edema	2.70	(1.81 − 3.99)	2.88	(1,92 − 4.31)	1.08	+2
Arthralgia or Arthritis	2.92	(1.81 − 3.99)	2.93	(2.10 − 4.17)	1.10	+2

95% CI: 95% confidence interval; OR: odds ratio;

* The final model included the clinical predictors listed above and days since onset of symptoms (≤ 3 / 4–7 days)

The area under the ROC curve of the derived clinical rule (Rio rule) was 69.5% (95% CI: 66.5−72.5), with an optimal cut-off point of 4 or higher, with 79.9% sensitivity and 51.0% specificity. The random split-half sample 2 (n = 1,606) showed 74.3% sensitivity and 51.5% specificity. Compared to estimates of previous clinical rules proposed by public agencies, the Rio rule included more symptoms and had higher specificity and positive likelihood ratio but lower sensitivity. The negative predictive value and negative likelihood ratio were similar to the estimates of the probable case definition adopted by the Brazilian Ministry of Health [21] and PAHO/CDC 2011 [7] (Table 3).

**Table 3:** Accuracy parameters for chikungunya case definitions by national and international health agencies, validation sample 2 (n = 1,606).

Clinical rules	Sens. %	Spec. %	PPV %	NPV %	LR+	LR-	DOR
	(95% CI)	(95% CI)	(95% CI)	(95% CI)	(95% CI)	(95% CI)	(95% CI)
Rio rule score ≥ 4 ^a^	74.3	51.5	26.1	89.7	1.53	0.49	3.07
	(69.0─79.2)	(48.8─54.3)	(23.1─29.1)	(87.3─91.8)	(1.41─1.67)	(0.41─0.61)	(2.32─4.08)
BRAZIL 2017 [21] or	81.6	41.2	24.2	90.7	1.39	0.44	3.12
PAHO/CDC 2011[7]^b^	(76.8─85.9)	(38.5─43.9)	(21.6─26.9)	(88.1─92.9)	(1.29─1.49)	(0.35─0.57)	(2.29─4.27)
WHO 2015 [32]^c^	82.0	39.8	23.8	90.6	1.36	0.91	3.01
	(77.2─86.2)	(37.1─42.5)	(21.3─26.5)	(87.9─92.8)	(1.27─1.46)	(0.88─0.93)	(2.20─4.12)
ECDC 2018 [33]^d^	96.3	6.7	19.2	88.8	1.03	0.54	1.89
	(93.5─98.2)	(5.4─8.2)	(17.2─21.3)	(80.9─94.3)	(1.01─1.06)	(0.29─1.01)	(1.00─3.59)

Sens: sensitivity; Spec: specificity; PPV: positive predictive value; NPV: negative predictive value; LR: likelihood ratio; DOR: diagnostic odds ratio.

BRAZIL: Brazilian Ministry of Health; PAHO: Pan American Health Organization; CDC: Centers for Disease Control; WHO: World Health Organization; ECDC: European Centers for Disease Control. ^a^ Rio rule score = fever*1 + exanthema* 1+ myalgia* 2+ arthralgia or arthritis* 2+ joint edema* 2; Probable case defined by ^b^ fever and (arthralgia or arthritis), ^c^ fever and arthralgia, ^d^ fever.

## Discussion

This study developed and validated a clinical rule for diagnosing chikungunya in a complex epidemiological scenario. Rio de Janeiro is the second largest Brazilian city and the fourth largest in Latin America, with 20% of its inhabitants living in slums with inadequate housing and sanitation. The city has a tropical climate (annual average temperature of 23.7°C) conducive to the proliferation of *Aedes aegypti*, with simultaneous circulation of dengue and Zika.

The best clinical criteria for diagnosing chikungunya include the presence of fever, exanthema, myalgia, arthralgia or arthritis, and joint edema. According to this rule, the presence of two joint symptoms suffices for clinical diagnosis of chikungunya, with a lower false-positive rate compared to the definitions proposed by WHO 2015 [32], PAHO/CDC 2011 [7], BRAZIL 2017 [21], and ECDC 2018 [33]. Adding more symptoms to the Rio rule improved specificity and positive likelihood ratio at the expense of lower sensitivity compared to definitions based on fever [33] or the combination of fever with arthralgia or arthritis [7, 21, 32]. The negative predictive value was around 90%, similar to the other definitions.

Fever and arthralgia were the most frequent symptoms in chikungunya cases in our study. Consistent with our findings, both predictors were included in a clinical rule derived and validated in a sample of patients 65 years or older (n = 687) from Martinique [37] The ECDC definition [33] would not be helpful in scenarios of arbovirus cocirculation since it is based exclusively on fever and could lead to high false-positive rates [23, 38].

Arthralgia and joint edema were the best predictors of chikungunya, consistent with other studies [6, 38–41]. The case definitions that include arthralgia, more subjective than joint edema, had the best sensitivity and were adequate to rule out chikungunya, with a negative predictive value of approximately 90%. This parameter was better than that obtained in a sample of 200 suspected cases in Jamaica (2014), of which 137 were serologically confirmed as chikungunya, showing a negative predictive value of 76.2% [42]. A study conducted in Southeast Africa also found 84% sensitivity with the WHO definition, with more promising specificity than ours [43].

In our study, myalgia and exanthema were more frequent in chikungunya cases compared to dengue and Zika. Although not included in national and international case definitions [7, 21, 32, 33], these symptoms were also statistically associated with chikungunya diagnosis in other studies in the Caribbean [44] and Brazil, the latter conducted in a proven scenario of dengue and Zika cocirculation [45].

Exanthema occurred in about one in three chikungunya cases, compared to one in five for dengue and one in six for Zika. This finding may be related to the fact that one-third of Zika cases sought health care after the third day since onset of symptoms. In Puerto Rico, where dengue is endemic, skin rash was also more frequent in adults with chikungunya compared to other febrile illnesses [40].

To our knowledge, this is the first study that derived and validated a clinical rule for chikungunya diagnosis in a large consecutive sample (3,214 patients seen in 23 primary and secondary healthcare facilities). The methodology followed the recommendations for validation studies [19, 30, 46].

In a sample of 687 patients admitted to acute healthcare services in Martinique, the derived clinical score included fever (3 points), ankle pain (2 points), lymphopenia (6 points), and absence of neutrophilia (10 points), where a score of 12 points or higher showed 87% sensitivity (83–90%) and 70% specificity (63–76%) [37]. However, the rule used non-specific laboratory parameters, which can hinder its use in resource-scarce settings. In a case-control derivation study [29] comparing 168 chikungunya and 452 dengue patients from French Guyana, joint (+5) and back pain (+1) were independently associated with chikungunya, while headache (-1), myalgia (-2), nausea/vomiting (-1), diarrhea (-1), and bleeding (-3) were associated with dengue.

Another strength of this study was the RT-qPCR gold standard applied to all suspected cases, with most serum samples collected within three days of onset of symptoms and after clinical evaluation. The RT-qPCR of the ZDC Molecular kit has shown 100% sensitivity and specificity [31], similar to the Trioplex kit of CDC [47]. Consistent with our results, joint pain, joint edema, skin rash, and muscle, bone, or back pain were significant predictors of chikungunya when compared to dengue or other acute febrile illnesses in a large sample from Puerto Rico using the same gold standard [40].

The study´s limitations include the sentinel surveillance data obtained from the Rio de Janeiro Municipal Health Department. A previous seroprevalence study estimated that the number of chikungunya cases could be at least 45 times higher than those reported to the surveillance system [13]. Although trained health professionals collected the data using standardized forms, they did not record bleeding manifestations or hematologic laboratory parameters, which are important for differential diagnosis with dengue. To deal with potential errors in medical evaluation, we combined arthritis and arthralgia in the analysis.

This study used the split-half internal validation approach. The large sample size allowed to derive and validate a clinical rule for diagnosing chikungunya in healthcare services normally used by patients with suspected arbovirus infections, such as primary care and urgent/emergency services. The samples did not show substantial imbalances in predictors or outcome distributions.

Our findings suggest that the best diagnostic clinical rule for acute-phase chikungunya diagnosis includes not only fever and joint symptoms such as pain and edema, but also exanthema and myalgia. This rule may lead the physician to order a confirmatory RT-qPCR for chikungunya diagnosis, which can be helpful in arbovirus surveillance in urban areas of dengue-endemic countries. Further studies should confirm the proposed diagnostic rule´s performance in other urban settings and evaluate bleeding as well as relevant hematologic parameters for differential diagnosis with dengue.

## Supporting information

S1 Table Distribution of clinical signs and symptoms according to diagnosis, Rio de Janeiro, Brazil, 2016–2019 (n = 3,214). 95% CI: 95% confidence interval OFI: Other febrile illness; b IQR: interquartile range.
(PDF)

S2 Table Odds ratio (OR) of clinical predictors according to chikungunya diagnosis (CHIK) in derivation (sample 1) and validation samples (sample 2). *p < 0.2; 95% CI: 95% confidence interval.
(PDF)
