# Supplementary material for: Development and validation of a clinical rule for the diagnosis of chikungunya fever in a dengue-endemic area
Source: PLoS One. 2023 Jan 6;18(1):e0279970. doi: 10.1371/journal.pone.0279970 (PMC9821784; doi:10.1371/journal.pone.0279970)
Supplement: S1 Table — 95% CI: 95% confidence interval OFI: Other febrile illness; b IQR: interquartile range. (PDF) [file pone.0279970.s001.pdf]

S1Table. Distribution of clinical signs and symptoms according to diagnosis, Rio de Janeiro, Brazil, 2016-2019 (n=3,214)

Variables	Category		Chikungunya (N=624)		Zika (N=88)		Dengue (N=51)		OFI [a] (N=2,451)
		n	% (95% CI)	n	% (95% CI)	n	% (95% CI)	n	% (95% CI)
Time since onset	≤ 3 days	519	83.2 (80.0 − 86.2)	58	65.9 (55.0 − 75.7)	38	74.5 (60.4 − 85.7)	1572	64.1 (62.2 − 66.0)
	4−7 days	105	16.8 (13.9 − 19.9)	30	34.1 (24.3 – 44.9)	13	25.5 (14.3 − 39.6)	879	35.9 (33.9 − 37.8)
Fever	Yes	600	96.2 (94.3 − 97.5)	85	96.6 (90.4 − 99.3)	46	90.2 (78.6 − 96.7)	2282	93.1 (92.0 − 94.1)
	No	24	3.8 (2.5 − 5.7)	3	3.4 (0.7 − 9.6)	5	9.8 (3.3 − 21.4)	169	6.9 (5.9 − 7.9)
Exanthema	Yes	179	28.7 (25.2 − 32.4)	13	14.8 (8.1 − 23.9)	11	21.6 (11.3 − 35.3)	580	23.6 (21.9 − 25.4)
	No	445	71.3 (67.6 − 74.8)	75	85.2 (76.1 − 91.9)	40	78.4 (64.7 − 88.7)	1871	78.4 (64.7 − 88.7)
Myalgia	Yes	482	77.2 (73.7 − 80.4)	59	67.0 (56.2 − 76.7)	14	27.5 (15.9 − 41.7)	1501	61.2 (59.3 − 63.3)
	No	142	22.8 (19.5 − 26.3)	29	33.0 (23.3 − 43.7)	37	72.5 (58.2 − 84.1)	950	38.8 (36.8 − 40.7)
Arthralgia	Yes	523	83.8 (80.7 − 86.6)	57	64.8 (53.8 − 74.6)	35	68.6 (54.1 − 80.9)	1570	64.1 (62.1 − 65.9)
	No	101	16.2 (13.4 − 19.3)	31	35.2 (25.3 − 46.1)	16	31.4 (19.1 − 45.9)	881	35.9 (34.0 − 37.9)
Arthritis	Yes	41	6.6 (4.8 − 8.8)	9	10.2 (4.8 − 18.5)	11	21.6 (11.3 − 33.3)	239	9.8 (8.6 − 10.9)
	No	583	93.4 (91.2 − 95.2)	79	89.8 (81.5 − 95.2)	40	78.4 (64.7 − 87.7)	2212	90.2 (89.0 − 91.4)
Joint edema	Yes	98	15.7 (12.9 − 18.8)	1	1.1 (0.2 − 6.2)	−	−	144	5.9 (4.9 − 6.8)
	No	526	84.3 (81.2 − 87.1)	87	98.9 (93.8 − 99.9)	51	100.0 (93.2 – 100.0)	2307	94.1 (93.1 − 95.0)

S1 Table. Continuation

Variables	Category		Chikungunya (N=624)		Zika (N=88)		Dengue (N=51)		OFI [a] (N=2,451)
		**n**	**% (95% CI)**	**n**	**% (95% CI)**	**n**	**% (95% CI)**	**n**	**% (95% CI)**
Limb edema	Yes	79	12.7 (10.2 − 15.5)	4	4.5 (1.3 − 11.2)	1	2.0 (0.5 − 10.4)	183	7.5 (6.5 − 8.6)
	No	545	87.3 (84.5 − 89.8)	84	95.5 (88.7 − 98.7)	50	98.0 (89.5 – 99.9)	2268	92.5 (91.4 − 93.5)
Headache	Yes	476	76.3 (72.7 − 79.6)	71	80.7 (70.9 − 88.3)	43	84.3 (71.4 − 92.9)	1927	78.6 (76.9 − 80.2)
	No	148	23.7 (20.4 − 27.3)	17	19.3 (11.7 − 29.1)	8	15.7 (7.0 − 28.6)	524	21.4 (19.8 − 23.1)
Retro-orbital pain	Yes	193	30.9 (27.3 − 34.7)	22	25.0 (16.4 − 33.4)	20	39.2 (25.8 − 53.9)	789	32.2 (30.3 − 34.1)
	No	431	69.1 (65.3 − 72.7)	66	75.0 (64.6 − 83.6)	31	60.8 (46.1 − 74.2)	1662	67.8 (65.9 − 69.7)
Conjunctival Hyperemia	Yes	52	8.3 (6.3 − 10.8)	3	3.4 (0.7 − 9.6)	5	9.8 (3.3 − 21.4)	154	5.6 (4.7 − 6.6)
	No	572	91.7 (89.2 − 93.7)	85	96.6 (90.4 − 99.3)	46	90.2 (78.6 − 96.7)	2314	94.4 (93.4 − 95.3)
Lymphadenopathy	Yes	7	1.1 (0.4 − 2.3)	−	−	1	2.0 (0.5 − 10.4)	40	1.6 (1.2 − 2.2)
	No	617	98.9 (97.7 − 99.5)	88	100.0 (95.8 − 100.0)	50	98.0 (89.5 − 99.9)	2436	99.3 (98.9 − 99.6)
Aphtha	Yes	7	1.1 (0.4 − 2.3)	1	1.1 (0.3 − 6.2)	−	−	15	0.6 (0.3 − 1.0)
	No	617	98.9 (97.7 − 99.5)	87	98.9 (93.8 − 100.0)	51	100.0 (93.0 − 100.0)	2436	99.4 (98.9 − 99.7)
Neurological Manifestations	Yes	1	0.2 (0.0 − 0.9)	1	1.1 (0.0 − 6.2)	1	2.0 (0.0 − 10.4)	22	0.9 (0.6 − 1.4)
	No	623	98.8 (99.1 − 99.9)	87	98.9 (93.8 − 99.9)	50	98.0 (89.5 − 99.9)	2429	99.1 (98.6 − 99.4)

95% CI: 95% confidence interval OFI: Other febrile illness; [b] IQR: interquartile range
