# Supplementary material for: Development and validation of a clinical rule for the diagnosis of chikungunya fever in a dengue-endemic area
Source: PLoS One. 2023 Jan 6;18(1):e0279970. doi: 10.1371/journal.pone.0279970 (PMC9821784; doi:10.1371/journal.pone.0279970)
Supplement: S2 Table — *p < 0.2; 95% CI: 95% confidence interval. (PDF) [file pone.0279970.s002.pdf]

S2 Table. Odds ratio (OR) of clinical predictors according to chikungunya diagnosis (CHIK) in derivation (sample 1) and validation samples (sample 2)

Variables		Sample 1 (n=1,608)						Sample 2 (n=1,606)					
		CHIK +		CHIK -				CHIK +		CHIK -			
		n	(%)	n	(%)	OR	95% CI	n	(%)		(%)	OR	95% CI
Time since onset	≤ 3	265	81.8	824	64.2	2.51	1.85 – 3.40	254	84.7	844	64.6	3.02	2.16–4.22
	4 to 7	59	18.2	460	35.8	1	−	46	15.3	462	35.4	1.00	−
Fever	Yes	311	96.0	1195	93.1	1.78*	0.98−3.23	289	96.3	1218	93.3	1.90*	1.00−3.60
	No	13	4.0	89	6.9	1	−	11	3.7	88	6.7	1.00	−
Exanthema	Yes	96	29.6	289	22.5	1.45*	1.10−1.90	83	27.6	315	24.1	1.20*	0.91−1.60
	No	228	70.4	995	77.5	1	−	217	72.3	991	75.9	1.00	−
Myalgia	Yes	255	78.7	768	59.8	2.48*	1.86−3.31	227	75.7	806	61.7	1.93*	1.45−2.57
	No	69	21.3	516	40.2	1	−	73	24.3	500	38.3	1.00	−
Arthralgia	Yes	272	83.9	842	65.6	2.75*	2.00−3.77	251	83.7	820	62.8	3.04*	2.19−4.21
	No	52	16.1	442	34.7	1	−	49	16.3	486	37.2	1.00	−
Arthritis	Yes	22	6.8	130	10.1	0.65*	0.40−1.03	19	6.3	129	9.9	0.62*	0.37−1.02
	No	302	92.2	1154	89.9	1	−	281	93.7	1177	90.1	1.00	−
Joint Edema	Yes	53	16.4	74	5.8	3.20*	2.19−4.66	45	15.0	71	5.4	3.07*	2.06−4.57
	No	271	83.6	1210	94.2	1	−	255	85.0	1235	94.6	1.00	−
Limb Edema	Yes	39	12.0	91	7.1	1.79*	1.21−2.67	40	13.3	97	7.4	1.92*	1.30−2.84
	No	285	88.0	1193	92.9	1	−	260	86.7	1209	92.6	1.00	−
Headache	Yes	246	75.9	998	77.7	0.90	0.68−1.20	230	76.7	1043	79.9	0.83	0.61−1.12
	No	78	24.1	286	22.3	1	−	70	23.3	263	20.1	1.00	−
Retro-orbital Pain	Yes	109	33.6	413	32.2	1.07	0.83−1.38	84	28.0	418	32.0	0.83	0.63−1.09
	No	215	66.4	871	67.8	1	−	216	72.0	888	68.0	1.00	−
Conjunctival Hyperemia	Yes	25	7.7	70	5.5	1.45*	0.90−2.33	27	9.0	75	5.7	1.62*	1.03−2.57
	No	299	92.3	1214	94.5	1	−	273	91.0	1231	94.3	1.00	−
Arthralgia or arthritis	Yes	277	85.5	860	67.0	2.91*	2.09-4.04	252	84.0	840	64.3	2.91*	2.10-4.05
	No	47	14.5	424	33.0	1	−	48	16.0	466	35.7	1.00	−

*p < 0.2; 95% CI: 95% confidence interval
